# The gut-skin axis in melanoma: from microbial regulatory mechanisms to clinical translation for precision management

**DOI:** 10.3389/fmicb.2026.1839030

**Published:** 2026-07-01

**Authors:** Xuanchi Su, Yang Xiang, Haoran Zhao, Ouyang Li, Ling Zhang, Baofeng Guo

**Affiliations:** 1 Department of Plastic Surgery, China-Japan Union Hospital of Jilin University, Changchun, China; 2 NHC Key Laboratory of Radiobiology, School of Public Health, Jilin University, Changchun, China; 3 The Second Norman Bethune Clinical Medical College of Jilin University, The Second Hospital of Jilin University, Changchun, China; 4 Key Laboratory of Pathobiology, Ministry of Education, Department of Biomedical Science, College of Basic Medical Sciences, Jilin University, Changchun, China; 5 The First Norman Bethune Clinical Medical College of Jilin University, The First Hospital of Jilin University, Changchun, China

**Keywords:** clinical translation, gut microbiota, gut-skin axis, melanoma, microbiota-host interaction biomarkers, precision microbiome modulation, skin microbiome

## Abstract

Melanoma is an aggressive cutaneous malignancy with poor prognosis in advanced stages. Immune checkpoint inhibitors (ICIs) act as first-line therapy, yet are limited by primary/acquired resistance and immune-related adverse events (irAEs). The gut-skin axis, which links gut and skin microbiota to host physiology, has been increasingly implicated in melanoma tumorigenesis, progression and therapeutic efficacy, while its systemic mechanisms and clinical value remain incompletely understood. In this review, the multi-dimensional regulation of melanoma via the gut-skin axis is dissected through six core axes, namely immune regulation, metabolism-tumor microenvironment, aging-inflammaging, endocrine, circadian rhythm and ultraviolet (UV) radiation. Mechanism-driven microbiota-host interaction biomarkers (MHIBs) are proposed as a hypothesis-generating framework, which differ from conventional biomarkers in aiming to capture functional microbiota-host crosstalk. Six microbiota-targeted intervention strategies and their potential synergistic effects with mainstream therapies including ICIs, targeted therapy, chemotherapy and radiotherapy are summarized alongside critical translational barriers, and a multi-omics-based framework for functional microbiota stratification is proposed. Notably, bidirectional gut-skin microbiota crosstalk is highlighted to conceptualize a working model of the “microbiota-gut-skin axis-melanoma” relationship, broadening the one-sided focus on gut microbiota. Key challenges in this field are addressed, including unclear causal relationships, lack of standardized research protocols and insufficient clinical evidence. Corresponding future research priorities are put forward for mechanistic validation, biomarker clinical translation and personalized intervention development, which provide novel insights for the precision diagnosis and treatment of melanoma.

## 1 Introduction

Melanoma is a highly aggressive cutaneous malignant tumor with a continuously rising global incidence (Ferlay et al., 2021). Its primary etiological factors include ultraviolet (UV) radiation and somatic mutations. While early-stage melanoma is curable via surgical resection, the survival rate of patients with advanced disease declines significantly. Notably, high-risk populations—including solid organ transplant recipients and individuals with a family history of melanoma—exhibit substantially elevated risks of both disease onset and mortality (Zwald et al., 2024; Jansen et al., 2025). Immune checkpoint inhibitors (ICIs), centered on PD-1/CTLA-4 inhibitors, have emerged as the first-line therapeutic regimen for advanced melanoma, which remarkably improves patient survival. However, primary/acquired resistance and immune-related adverse events (irAEs) remain major clinical bottlenecks (Hodi et al., 2010; Tawbi et al., 2022).

As the host’s “second genome,” the gut microbiota contributes to physiological homeostasis through immune and metabolic modulation. While the compositional features of dysbiosis are highly heterogeneous across different diseases, under conditions of intestinal mucosal inflammation or epithelial barrier dysfunction, it is frequently characterized by an expansion of facultative anaerobes, particularly within the phylum *Proteobacteria* (Shin et al., 2015). Within the gut-skin axis model, gut microbiota alterations have been associated with melanoma initiation, progression, and treatment response. In selected preclinical and clinical contexts, the microbiota may influence melanoma therapy through pathways involving tumor microenvironment (TME) immune infiltration (Abdullah et al., 2024), microbial metabolite translocation (Zhuang et al., 2024), and systemic inflammatory status (Escorcia Mora et al., 2025).

In contrast to the gut microbiota, the role of the skin microbiota in melanoma tumorigenesis and ICIs response remains incompletely elucidated. Commensal skin bacteria potentiate cutaneous immunity to protect against cutaneous infections, inflammatory disorders, and malignant neoplasms (Nakatsuji et al., 2018; Stacy and Belkaid, 2019). The skin microbiota may undergo dysbiosis in response to damaging insults (Dong et al., 2022). For instance, in the MeLiM (Melanoma-bearing Libechov Minipig) porcine model, *Fusobacterium*, *Trueperella*, *Staphylococcus*, *Streptococcus*, and *Bacteroides* have been reported to be enriched in the intratumoral microbiome of melanoma (Mekadim et al., 2022). The skin microbiota augments host innate immunity via activation of pattern recognition receptors (PRRs), including Toll-like receptor 2 (TLR2) and Toll-like receptor 4 (TLR4). Conversely, sustained activation of TLRs is linked to chronic inflammation and carcinogenesis (Park et al., 2024).

In this review, we synthesize the core regulatory mechanisms that link the gut-skin axis to melanoma, critically evaluate the clinical utility and limitations of microbiome biomarkers, summarize advances in microbiota-targeted interventions, and propose a multi-omics-based framework for future precision-management studies. Crucially, this review differs from prior literature that primarily focuses on the unidirectional gut-tumor axis. By integrating the bidirectional microbiota-gut-skin network, we incorporate forward-looking, underexplored variables into our framework. These include aging, circadian rhythms, sex-specific endocrine signaling, and the cutaneous UV resistome.

## 2 The role of the microbiota in the initiation and progression of melanoma

### 2.1 Microbiota in melanoma initiation and progression

The relationship between the commensal microbiota and melanoma can be considered from two complementary perspectives: the tumor-suppressive or pro-tumorigenic effects of specific bacterial taxa, and the dynamic alterations of the gut microbiota across the trajectory of tumor progression. Early investigations in this field have predominantly centered on the analysis of the taxonomic composition and functional phenotypes of the gut microbiota in patients with melanoma.

Substantial compositional differences in the gut microbiota have been reported between melanoma patients and healthy controls, with microbial signatures shifting dynamically across advancing tumor stages. Multiple studies have documented significant enrichment of *Prevotella copri* and yeast populations in patient cohorts (Vitali et al., 2022). Specifically, early-stage melanoma is characterized by an enrichment of immunomodulatory commensal taxa, whereas advanced disease is associated with reduced microbial diversity and a marked expansion of pro-inflammatory bacterial genera (Witt et al., 2023; Björk et al., 2024). Aberrant microbial profiles have also been identified across the gut mycobiota, bacterial community assemblages, and skin microbiota of melanoma patients, yet systematic investigations into these multi-kingdom, multi-compartment microbial alterations remain sparse.

In preclinical models and selected clinical studies, specific commensal gut bacteria suppress melanoma progression primarily via two distinct mechanistic paradigms. First, some taxa can enhance systemic anti-tumor immunity in Rnf5(−/−) mice; for example, specific *Corynebacterium* strains have been reported to induce anti-tumor immunity that restricts tumor outgrowth (Li et al., 2019), while short-chain fatty acid (SCFA)-producing bacterial genera inhibit melanoma bone metastasis (Pal et al., 2022). Second, microbiota modulation augments immunotherapeutic responses: fecal microbiota transplantation (FMT) from donors with durable ICIs responses can restore therapeutic reactivity to PD-1 inhibitors in patients with refractory disease, with underlying mechanisms tightly linked to the expansion of immunoactive commensal taxa and enhanced activation of CD8^+^T cells (Davar et al., 2021; Rebeck et al., 2021). Conversely, gut microbiota depletion via antibiotic administration accelerates melanoma progression (Davar et al., 2021).

To date, the research focus in this field has shifted from phenotypic profiling of the microbiota to in-depth mechanistic dissection, with investigative directions including the microbiota-immune axis (Pal et al., 2022), microbial metabolite-mediated modulation of TME (Chen et al., 2021), and FMT in combination with immunotherapy (Davar et al., 2021; Liu et al., 2025). The functional mediators of the aforementioned regulatory mechanisms are specific intestinal and skin bacterial and fungal taxa closely associated with melanoma tumorigenesis, progression and therapeutic response. We systematically summarize the action mechanisms, therapeutic applications and key supporting references of these well-validated core species in Table 1.

**Table 1:** Characterization of intestinal and skin microorganisms in melanoma progression and therapeutic response.

Microorganism species	Primary source	Core function in melanoma	Key effector mechanisms	Therapeutic relevance	References
*Akkermansia muciniphila*	Intestinal tract	Anti-tumor immune regulation; opportunistic colitis induction	STING-IFN-I pathway activation; TLR2-TLR1 innate immunity modulation; abundance correlates with ICI response	Modulates ICI clinical outcomes in melanoma	Seregin et al. (2017), Gopalakrishnan et al. (2018), Routy et al. (2018), Lam et al. (2021), Bae et al. (2022), Shi M. et al. (2022)
*Faecalibacterium prausnitzii*	Intestinal tract	Anti-tumor effect; melanoma recurrence inhibition; radiosensitizer	Inhibits recurrence-driving lipid metabolites; butyrate-induced cancer cell autophagy	Improves 2-year RFS in stage IIIB/C melanoma; exerts radiosensitizing effect	Teng et al. (2023), Alves Costa Silva et al. (2024)
*Bifidobacterium* spp. (including *B. longum* and *B. angulatum*)	Intestinal tract	Activates anti-tumor immunity; enhances systemic anti-tumor responses	DC and CTL activation; reduces tumor-infiltrating Tregs; increases CD8^+^IFN-γ^+^ T cells	Synergizes with oncolytic adenovirus Ad-CpG; retards melanoma progression	Tripodi et al. (2023), Chen Z. et al. (2025)
*Lactobacillus reuteri*	Intestinal tract, melanoma tissues	Anti-tumor effect; enhances CD8^+^ T cell cytotoxicity	Tryptophan metabolism to I3A; activates AhR-CREB axis in CD8^+^ T cells	Significantly improves ICI therapy efficacy	Bender et al. (2023)
*Enterococcus faecalis*	Intestinal tract, melanoma tissues	Anti-tumor effect; enhances ICI therapy efficacy	NOD2 pathway activation; STING-IFN-I innate immunity activation; enhances DC function	Synergizes with anti-PD-1/PD-L1 ICIs; overcomes anti-PD-1 resistance	Griffin et al. (2021), Xian et al. (2025)
*Roseburia* spp.	Intestinal tract	Maintains gut microbiota homeostasis; anti-tumorigenesis	SCFAs production; sustains intestinal microbiota balance	Reduced abundance in melanoma; accompanied by *Fusobacterium* enrichment	Charbel et al. (2025)
*Malassezia* spp., *Candida albicans*, *Candida dubliniensis* and other fungi	Intestinal tract	Dual ICI response effects; modulates melanoma progression risk	Regulates intestinal mycobiota structure; modulates systemic anti-tumor immunity	Abundance correlates with treatment outcomes; low richness links to positive ICI response	Szóstak et al. (2024)
*Ruminococcus* spp. (e.g., strain YB328)	Intestinal tract, TME	Anti-tumor effect; enhances immunotherapy efficacy	Activates intestinal DCs; drives tumor-specific CD8^+^ T cell activation	Strain YB328 enhances anti-PD-1 inhibitor efficacy	Lin N. Y. et al. (2025)
*Staphylococcus epidermidis*	Skin commensal bacterium, distal tumor tissues	Dual effects; core anti-tumor activity; pro-survival metabolite production	Induces tumor-specific CD8^+^ T cells; metabolite 6-HAP exerts anti-tumor effect	Synergizes with ICIs; reverses “cold tumor” ICI insensitivity	Chen et al. (2023), Charbel et al. (2025)
*Bacteroides* spp.	Intestinal tract	Dual effects; anti-tumor immune activation; pro-inflammatory tumor promotion	cGAS-STING pathway activation; menaquinone/LPS-driven chronic inflammation	Beneficial strains enhance ICI efficacy; some strains impair anti-PD-1 response	Björk et al. (2024), Peng K. et al. (2025)
*Corynebacterium* spp.	Acral melanoma lesions	Promotes tumor progression	Correlates with IL-17^+^ cell infiltration; activates IL-6/STAT3 pathway	Increased abundance in advanced acral melanoma	Charbel et al. (2025)

### 2.2 Mechanisms of microbiota action in melanoma tumorigenesis and progression

#### 2.2.1 Immune regulatory axis

Immune evasion is a core driver of melanoma progression and metastatic dissemination. Diverse components within the melanoma TME exert critical modulatory roles in this process. These components mainly include cellular compartments such as CD8^+^ T cells (Kleffel et al., 2015; Mo et al., 2018), regulatory T cells (Tregs) (Ataera et al., 2011), myeloid-derived suppressor cells (MDSCs) (Poschke et al., 2010; Weide et al., 2014), and tumor-associated macrophages (TAMs) (Tcyganov et al., 2018), as well as non-cellular elements like exosome-containing extracellular vesicles (EVs) (Hood et al., 2011; Chen G. et al., 2018; Poggio et al., 2019) and environmental triggers represented by UV radiation (Shreedhar et al., 1998). Modulating microbiota-immune interactions may offer therapeutic opportunities, but the degree to which these interactions can be targeted clinically remains under investigation.

**Figure 1:**
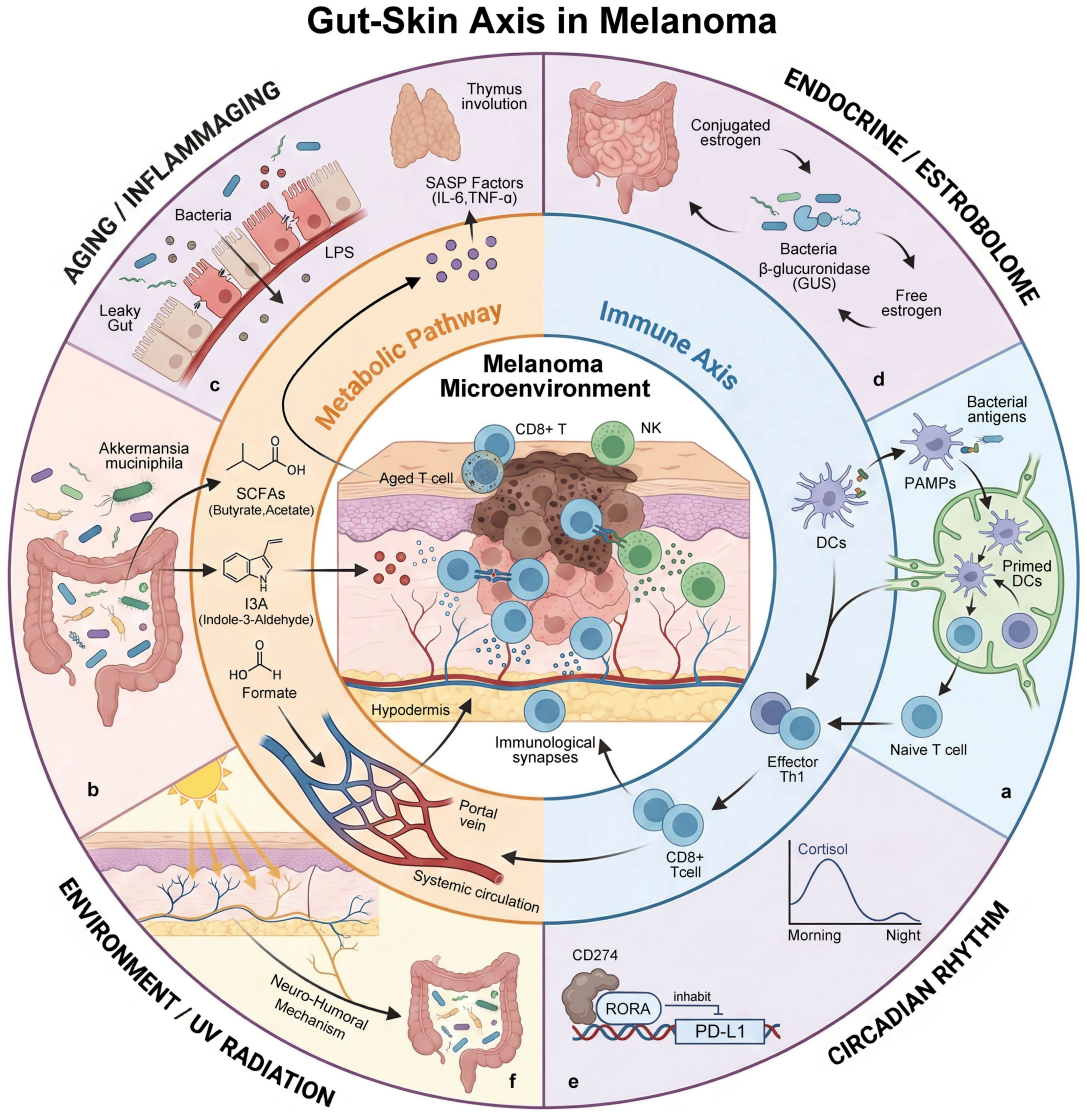
Schematic overview of the gut-skin axis in melanoma. This figure depicts the core regulatory axes of the gut-skin axis linking gut/skin microbiota, host physiology and the TME. **(A)** Microbiota-immune axis: Microbial antigens activate dendritic cells to drive effector T cell anti-tumor immunity and modulate ICIs response. **(B)** Microbiota-metabolism-TME axis: Commensals (e.g., *Akkermansia muciniphila*) produce SCFAs, I3A and formate to systemically remodel the TME. **(C)** Aging-inflammaging axis: Gut barrier disruption drives LPS translocation and SASP factor (IL-6, TNF-α) release to form a pro-tumorigenic inflammatory milieu. **(D)** Endocrine axis: GUS regulates host systemic free estrogen levels. **(E)** Circadian rhythm axis: Physiological cortisol diurnal oscillations sustain RORA expression, which inhibits PD-L1 to en-hance anti-tumor immunity and ICI efficacy. **(F)** UV radiation axis: Cutaneous UV exposure drives parallel skin and gut microbiota changes via neuro-humoral mechanisms. Created with BioRender.com.

The gut microbiota shapes systemic immune status in the host via modulating both innate and adaptive immunity (Aghamajidi and Maleki Vareki, 2022; Li et al., 2023), and is implicated in orchestrating immune surveillance and the modulation of immunotherapeutic responses in melanoma (Figure 1A). At the innate immune level, commensal microbiota initiate immune responses via the pathogen-associated molecular patterns (PAMPs)-PRRs axis. Microbial metabolite SCFAs reinforce intestinal barrier integrity (Xu et al., 2013; Liu T. et al., 2023), and attenuate inflammation in both the intestinal tract and distal TME via inhibiting the nuclear factor kappa-B (NF-κB) signaling pathway (Gill et al., 2018). At the adaptive immune level, the gut microbiota modulates the differentiation and homeostatic balance of T cell subsets (Round et al., 2011; Flannigan and Denning, 2018), activates dendritic cells (DCs), and regulates B-cell differentiation (Li H. et al., 2020). Immune cells can also reciprocally shape gut microbial composition, forming a bidirectional feedback loop (Gu et al., 2024).

The gut and intratumoral microbiota influence melanoma through local and systemic immune landscapes. Melanomas with abundant intratumoral T cell infiltration and features of spontaneous regression exhibit high immunogenicity and enhanced sensitivity to immunotherapies (Lattanzi et al., 2024). Conversely, tumor cells can achieve immune “camouflage” through multiple mechanisms including defects in chemokine secretion, which restricts immune cell infiltration into TME and confers a poor clinical prognosis (Daillère et al., 2020). Current evidence suggests that intratumoral-resident microbiota are associated with local chemokine expression and CD8^+^ T cell infiltration, features linked to survival outcomes in cutaneous melanoma (Zhang et al., 2022). Beneficial commensal taxa, including *Akkermansia muciniphila*, synergize with PD-1 inhibitors to augment anti-tumor immunity and restrain melanoma outgrowth (Wu et al., 2025).

Melanoma is characterized by high invasiveness and metastatic propensity, and the commensal microbiota contribute to the metastatic cascade via immune regulatory pathways. The microenvironment of metastatic niches remains the critical “soil” in this process, consistent with the “seed and soil” theory of tumor metastasis. Emerging evidence indicates that the gut microbiota can modulate apolipoprotein E (ApoE) signaling (Seo et al., 2023). Given that ApoE is a critical regulatory mediator of melanoma invasion and metastasis (Pencheva et al., 2012; More et al., 2024), we propose, as a testable hypothesis, that the gut microbiota may shape melanoma metastatic potential partly by modulating host ApoE expression or activity. ApoE may represent an underexplored immunometabolic node within the gut-skin axis, but melanoma-specific causal validation remains lacking. Importantly, these systemic immune modulations do not occur in isolation; rather, gut microbiota-derived metabolites often function as the critical paracrine messengers bridging these spatial barriers.

Evidence for the immune regulatory axis is strongest for melanoma-specific human associations with ICIs response and for early interventional FMT studies, but many mechanistic claims still rely on murine models or *in vitro* systems.

#### 2.2.2 Microbiota-metabolism-TME axis

Gut microbiota-derived metabolites may serve as intermediary signals linking commensal microbes to melanoma biology, primarily through remodeling the TME and modulating signaling pathways in malignant cells and immune cell subsets (Yang et al., 2023) (Figure 1B). The TME exerts multifaceted, context-dependent effects on the therapeutic efficacy of systemic therapies. Distinct visceral metastatic melanoma lesions possess unique immune microenvironment signatures (Conway et al., 2022). Consequently, targeting the microbiota-metabolism-TME axis represents a highly promising avenue for melanoma intervention.

Formate, a gut microbiota-derived metabolite, augments the effector function of CD8^+^ T cells via Nrf2 signaling pathway, thereby mediating exercise-associated anti-tumor effects and improved ICIs efficacy (Phelps et al., 2025). These findings support formate as a promising biomarker candidate, but its predictive value requires prospective clinical validation.

Microbial metabolites, such as SCFAs, are essential for maintaining intestinal barrier integrity (LeBlanc et al., 2013). During states of barrier dysfunction, which are frequently observed in systemic inflammation or infectious diseases, the gut microbiota can translocate to mesenteric lymph nodes or distant organs, including tumor tissues (Ubeda et al., 2010; Ayres et al., 2012; Zeng et al., 2016; Peyrin-Biroulet et al., 2012). While this translocation is known to reprogram the TME, its precise causal role in human melanoma initiation, progression, and therapeutic response remains to be fully elucidated. Systemic administration of *Bifidobacterium* results in its accumulation within tumors, converting anti-CD47 immunotherapy non-responders to responders in mouse models (Shi et al., 2020). Notably, the causal relationship between the microbiota in the TME and immunotherapeutic efficacy may be reversed in certain contexts. ICIs can induce lymph node remodeling and DC activation, selectively promoting the translocation of intestinal bacterial subsets to extraintestinal tissues, which potentiates anti-tumor T cell responses in tumor-draining lymph nodes (TDLNs) and primary tumors (Choi et al., 2023). Furthermore, intratumoral microbiota can activate host anti-tumor immunity via antigen presentation, and their signatures hold potential as predictive biomarkers for therapeutic responses in melanoma (Kalaora et al., 2021; Wu et al., 2023).

SCFAs are produced by the gut microbiota via fermenting polysaccharides (Mirzaei et al., 2021) and exert context-dependent immune effects (Arpaia et al., 2013; Smith et al., 2013; Luu et al., 2021). Human and preclinical studies suggest that SCFAs are differentially associated with the efficacy of anti-PD-1 (Chaput et al., 2017; Frankel et al., 2017) and anti-CTLA-4 therapies (Coutzac et al., 2020). The tryptophan metabolite indole-3-acetic acid (I3A) enhances tumor cell immunogenicity and T cell activation, and its serum levels are closely associated with ICIs response in melanoma patients (Cui et al., 2025). Serum I3A levels are higher in melanoma patients who respond to ICIs than in non-responders (Bender et al., 2023), underscoring its potential value as a prognostic biomarker for immunotherapy in melanoma. Interestingly, intestinal *Lactobacillus* metabolizes tryptophan into indole, which reduces the number of anti-tumor CD8^+^ T cells and promotes the growth of pancreatic tumor cells (Hezaveh et al., 2022). These divergent findings illustrate the double-edged and context-dependent role of microbial metabolites. Other metabolites, including secondary bile acids, creatinine, polyamines, and arginine, may alter TME immunity by affecting cytokine production, T-cell and NK-cell proliferation, or Treg differentiation (Davar et al., 2021; Rebeck et al., 2021; Kumar et al., 2022; Simpson et al., 2022; Vitali et al., 2022; Fortman et al., 2023; Liu et al., 2025).

The skin microbiota may influence melanoma biology through microbial metabolites and local immune signaling. For example, skin microbiota-derived SCFAs can suppress histone deacetylase (HDAC) activity and modulate pathways involved in melanoma-cell proliferation, invasion, and migration (Sanford et al., 2016; Zheng et al., 2025). Mendelian randomization studies have established a causal link between the skin microbiota and melanoma (Zhu et al., 2024). Notably, bacteria from the same genus can exert opposing effects on melanoma development. For instance, *Staphylococcus aureus* induces DNA damage in melanocytes, whereas *Staphylococcus epidermidis* suppresses early mutated melanocytes (Fortman et al., 2023; Liu et al., 2025). To date, the therapeutic value of skin microbiota-targeted interventions in melanoma remains largely unexplored.

The metabolism-TME axis is supported by mechanistic studies and selected melanoma cohort data, but metabolite effects are highly context-dependent.

#### 2.2.3 Host physiological axis

##### 2.2.3.1 Aging

The composition of the gut microbiota undergoes continuous alterations with advancing age, and dietary patterns (Wang Y. et al., 2024; Theis et al., 2025), feeding and circadian rhythms (Peng T. et al., 2025), as well as smoking and alcohol consumption (Adhikary et al., 2024), all exert profound effects on immune homeostasis and cellular senescence. Intestinal barrier dysfunction, a highly prevalent condition in aged individuals, serves as a critical intermediary link connecting aging to a wide spectrum of diseases, including melanoma. The translocation of bacteria and their derived metabolites triggers systemic inflammation, which in turn accelerates the progression of neurodegenerative and other age-related disorders (Sharma, 2022; Shi X. et al., 2022).

Current paradigms suggest that the microbiota does not merely mirror chronological aging, but may actively contribute to age-related physiological declines, notably immunosenescence. Preclinical models support this concept; for instance, FMT from aged donors to young recipients is sufficient to induce systemic inflammatory phenotypes (Thevaranjan et al., 2017). Concurrently, specific microbial metabolites have been implicated in promoting cellular senescence in host cells (Yang et al., 2025), with reduced abundance of *Akkermansia* and diminished butyrate synthesis pathways reported as hallmarks of aging (Shin et al., 2021).

Immunosenescence refers to the progressive functional decline of the immune system with advancing age (Liu Z. et al., 2023), a process that shapes the initiation and progression of tumors. The central role of the γδ17 cell-neutrophil-CD8^+^ T cell immunoregulatory axis in aging-driven melanoma metastasis has been well characterized (Duan et al., 2024). With advancing age, the functional deterioration of immune organs and the accumulation of senescent T cells give rise to a senescence-associated secretory phenotype (SASP). Through this phenotype, cells release a broad spectrum of soluble mediators (e.g., IL-6 and TNF-α) that collectively establish a chronic pro-inflammatory milieu tightly linked to tumorigenesis and tumor invasion (Figure 1C).

Probiotic and prebiotic interventions in older adults can partially restore gut microbiota homeostasis (Salazar et al., 2017; Parker et al., 2022), but their relevance to melanoma-specific outcomes remains uncertain. Aging reshapes the frequency and functional status of diverse immune cell populations, thereby establishing an immune microenvironment network that drives tumorigenesis and metastasis. However, aging does not exert solely immunosuppressive effects on tumor immunity. Within the TME, cellular senescence and the SASP critically modulate local immunity (Kaur et al., 2016; Marin et al., 2023). Therefore, future microbiome-oncology studies must carefully delineate whether microbial signals primarily influence systemic immunosenescence, or directly impact localized cancer-cell senescence.

Aging-related microbiome mechanisms are biologically plausible but remain incompletely validated in melanoma patients. Preclinical transfer experiments support causality for inflammatory phenotypes, whereas clinical relevance for melanoma progression and treatment response remains mostly inferential.

##### 2.2.3.2 Endocrine

Gut dysbiosis can perturb hormonal homeostasis, suggesting that the microbiota functions as an active participant in the systemic endocrine network (Lin D. et al., 2025; Basnet et al., 2024). Both the gut microbiota and the intestinal tract possess the capacity to synthesize hormone-like bioactive molecules, establishing them as a bona fide endocrine organ in the broad sense. For instance, specific intestinal bacterial taxa secrete β-glucuronidase (GUS), an enzyme that modulates systemic estrogen levels *in vivo* (Baker et al., 2017; Pellock and Redinbo, 2017) (Figure 1D). During dysbiosis, aberrant GUS activity may increase circulating free estrogen, a pathway that has been associated with tumorigenesis in preclinical settings (Flores et al., 2012; Chen and Madak-Erdogan, 2016).

Circadian clock disruption is frequent in carcinogenesis and may promote tumor progression by influencing malignant growth and immune microenvironment remodeling. Dysregulation of circadian clock genes is well documented in melanoma, and is linked to immune escape (Liu et al., 2024). In murine melanoma models, the circadian oscillations of serum cortisol levels are abrogated (Aiello et al., 2022) (Figure 1E). Tumor-infiltrating CD8^+^ T cells exhibit circadian oscillations in both cell number and phenotypic profile, a process driven by the endogenous circadian clock of leukocytes and that of endothelial cells within the TME (Wang C. et al., 2024). The clock-dependent regulation of anti-tumor immunity enables the identification of the optimal administration timing for ICIs therapy (Fortin et al., 2024). Both high peak corticosteroid doses and second-line immunosuppressive agents for the management of irAEs are associated with impaired survival outcomes (Verheijden et al., 2024). For patients with melanoma, appropriate modulation of circadian rhythms is essential to restrain the inflammatory state and preserve anti-tumor immune function, but lifestyle or chronotherapy recommendations require prospective testing.

Sex-specific manifestations of melanoma are characterized by pronounced differences in incidence, metastatic patterns, and treatment response between male and female patients (Raymond et al., 2025). Reduced E-cadherin expression has been shown to upregulate the expression of estrogen receptor α (ERα), a pathway that may promote distant metastasis of melanoma in preclinical models. This sex-specific, estrogen-sensitizing mechanism provides a potential explanation for the observation that premenopausal women have a markedly higher incidence of melanoma than age-matched male counterparts (Meierjohann, 2025; Raymond et al., 2025). Older women exhibit lower circulating levels of bone morphogenetic protein 2 (BMP2), whereas senescent dermal fibroblasts from older men have been shown to specifically secrete BMP2. In preclinical models, this sex-specific secretion upregulates the expression of invasion-associated genes in melanoma cells, thereby augmenting the metastatic potential of tumors. Inhibiting BMP2 activity may partially reverse the invasive phenotype of melanoma in older men, offering a potential therapeutic avenue (Chhabra et al., 2024). Male patients with cutaneous melanoma achieve higher overall survival following ICIs therapy than female patients (Haupt et al., 2021). MHC-dependent selection of driver mutations is most prominent in young patients, with the strength of MHC class II-mediated selection in young women being nearly twice that observed in age-matched young men (Castro et al., 2020).

Endocrine, sex, and circadian pathways may modify melanoma immunity, but melanoma-specific microbiome data are sparse. These sections should therefore be interpreted as mechanistic context and hypothesis generation rather than established microbiota-based therapeutic guidance.

### 2.3 Anatomical and signaling basis of the gut–skin axis

Both the intestinal tract and the skin are extensively vascularized and innervated, and share overlapping immune and neuroendocrine networks including vagal reflexes and enteropeptide signaling (Salem et al., 2018). Environmental cues such as UV radiation exert not only direct effects on the skin, but also indirect impacts on distant organs including the intestinal tract, with microbiota alterations representing one possible mediator (Figure 1F). Exposure to UV radiation of specific frequencies and wavelengths increases the diversity of the gut microbiota (Bosman et al., 2019). The intestinal tract harbors a microbial community dominated by the phyla Bacteroidetes and Firmicutes, while the skin hosts a microbial community primarily composed of genera including *Staphylococcus* and *Propionibacterium* (Mahmud et al., 2022).

Immune system-mediated crosstalk is widely present between the gut and the skin. The gut microbiota activates DCs, mucosal-associated invariant T (MAIT) cells and other immune cell populations via the TLR and NF-κB signaling pathways, thereby regulating T cell homeostasis (Mahmud et al., 2022). Metagenomic sequencing has revealed a signature of reduced abundance of *Eubacterium rectale* and its associated functional genes in patients with psoriasis (Xiao Y. et al., 2024), supporting a role for gut microbiota in immune-mediated skin disease. Direct evidence supporting the existence of the gut–skin axis has also been observed: in murine models, cutaneous injury drives a skin-to-gut axis where systemic inflammatory mediators released from the dermis disrupt intestinal immune homeostasis and ultimately alter the gut bacterial community (Dokoshi et al., 2024).

Gut microbiota-derived metabolites modulate skin function via the systemic circulation, while the skin conversely exerts regulatory effects on the intestinal tract through mediators including vitamin D and tryptophan metabolites (Fletcher et al., 2023). Homeostasis of the skin microbiota is likewise indispensable for intestinal immunity and even the systemic immune homeostasis of the whole organism, whereas melanoma-associated microbiota dysbiosis can drive intestinal barrier damage and exacerbated inflammation via feedback signaling along the gut–skin axis. In the setting of cutaneous microbial dysbiosis, pathogens such as *Propionibacterium acnes* can trigger local inflammatory responses. The high levels of interleukin-17 (IL-17) released in this process disseminate to the intestinal tract via the systemic circulation, activating the Th17 cell axis in the intestinal lamina propria and thereby disrupting intestinal mucosal integrity (L'Orphelin et al., 2025). While this proposed mechanism outlines an intriguing theoretical feedback loop along the gut-skin axis, its direct relevance to melanoma progression necessitates empirical validation.

While microbial communities help maintain intestinal barrier integrity (LeBlanc et al., 2013), disruption of this barrier allows gut bacteria and their metabolites to enter the systemic circulation, a state known as “leaky gut” (Szántó et al., 2019). This systemic translocation, alongside the migration of gut-activated T cells and the release of enteroendocrine peptides, collectively propagates distant inflammatory responses in the skin. Clinically, this gut-skin crosstalk is evidenced in acral melanoma, where cutaneous dysbiosis frequently coincides with elevated intestinal inflammatory markers (e.g., fecal calprotectin) and local IL-17 overexpression (Gui et al., 2022). UV radiation exerts complex effects by altering the cutaneous microbiome and originating a “UV resistome”—a microbial adaptation to solar stress. Current consensus indicates this local adaptation plays a dual role in skin cancer initiation; furthermore, it is hypothesized that such UV-driven cutaneous perturbations could systematically modulate intestinal immune homeostasis via the gut-skin axis. However, the precise mechanisms and directionality of this distant cross-talk remain largely theoretical. Patients with multiple primary melanoma frequently present with concurrent cutaneous microbiota dysbiosis and intestinal dysfunction, which is associated with recurrent disease onset (Palacios-Diaz et al., 2022).

Gastrointestinal metastasis of cutaneous melanoma illustrates the anatomical connection between skin-derived malignancy and the intestinal environment. Melanoma is among the most common tumors that metastasize to the gastrointestinal tract (Lee et al., 2019), yet the mechanisms mediating the high gastrointestinal organotropism of melanoma remain poorly understood. Patients with non-melanoma skin cancer (NMSC) have an elevated risk of melanoma-related death, which indirectly supports that the metastatic potential of primary tumors is exacerbated under the inflammatory background of the gut–skin axis (Chen S. T. et al., 2018).

At present, the microbiota-dependent interaction mechanisms within the gut–skin axis have been relatively well characterized, while the mechanisms underlying non-microbiota-dependent mediators and the reverse skin-to-gut crosstalk remain to be further elucidated.

### 2.4 Microbiota and its metabolites as biomarkers

Histopathological examination remains the gold standard for the clinical diagnosis, prognostic stratification, and treatment decision-making of melanoma, while emerging biomarkers have further improved the predictive efficacy for disease recurrence (Aung et al., 2025). Lactate dehydrogenase (LDH) is a classic serological biomarker incorporated into the AJCC staging system. Dynamic alterations in circulating tumor DNA (ctDNA) are closely associated with the therapeutic efficacy of ICIs (Ho et al., 2026). The combined detection of circulating tumor cells (CTCs) and protein biomarkers such as S100B possesses higher clinical value than single-biomarker assays (Sawerska et al., 2025). Conventional microbiome-based biomarkers typically rely on the relative abundance of a single bacterial species. Such metrics are highly susceptible to inter-individual heterogeneity and often merely reflect statistical correlations rather than the underlying mechanisms driving the disease.

To address these limitations, we propose the theoretical framework of “mechanism-driven microbiota-host interaction biomarkers” (MHIBs). Unlike conventional 16S rRNA sequencing-based biomarkers that often capture only static taxonomic abundance, ideal MHIBs should be able to reflect the biochemical and immunological outputs of the microbiota. MHIBs are preliminarily defined as specific products jointly produced by bidirectional biochemical or immunological crosstalk between the host and the microbiota, such as co-metabolites or immune cells educated by specific microbiota. Serving as signaling mediators bridging the microecology and the host, MHIBs directly participate in modulating the systemic immune status and local tumor microenvironment. Compared to conventional biomarkers, MHIBs aim to more accurately reflect the mechanisms that potentially mediate tumor evolution, immune evasion, and therapeutic response, thereby indicating patient prognosis and treatment benefit.

Given that this field is still in its early developmental stages, it is necessary to explicitly distinguish data based on human melanoma clinical cohorts from extrapolated data derived from preclinical models or other cancer types. In human melanoma clinical cohorts, I3A exhibits potential as a prognostic MHIB. The serum levels of I3A are elevated in immunotherapy responders, which may exert effects by enhancing tumor cell immunogenicity and T cell activation (Bender et al., 2023). Similarly, gut-derived formate has been validated in the context of human melanoma to augment the effector functions of CD8^+^ T cells via the Nrf2 signaling pathway, representing a highly promising predictive biomarker (Phelps et al., 2025).

On the other hand, some candidate MHIBs with relatively in-depth investigations currently rely primarily on the support of preclinical models or pan-cancer data. For instance, secondary bile acids are archetypal co-metabolites synthesized by the host liver and enzymatically modified by gut microbiota. Recent murine melanoma model studies demonstrate that specific microbiota-derived secondary bile acids (e.g., 3-oxo-Δ4,6-LCA) can act as antagonists to the human androgen receptor (AR), enhancing the efficacy of anti-PD-1 therapy by blocking AR signaling within CD8^+^ T cells (Jin et al., 2025). At the immunological level, *in vivo* murine models have confirmed that intestinal colonization by segmented filamentous bacteria (SFB) can educate and induce phenotypic plasticity in specific clonotypic T cells, prompting their migration into the melanoma microenvironment to exert targeted cytotoxic effects (Najar et al., 2026). Furthermore, in human pan-cancer settings (such as renal cell carcinoma and non-small cell lung cancer), soluble mucosal addressin cell adhesion molecule 1 (sMAdCAM-1) has been proposed as a composite biomarker reflecting gut microbial dysbiosis. In these contexts, antibiotic-induced expansion of *Enterocloster* species was shown to alter bile acid profiles and downregulate host MAdCAM-1 expression, which correlated with poorer immunotherapy outcomes (Jin et al., 2025). Given the unique immune contexture of the skin, the extrapolation of these pan-cancer mechanistic links to melanoma warrants cautious interpretation and requires specific validation in prospective cohorts.

Despite their potential translational value, applying the MHIBs framework to routine clinical practice still faces methodological and biological hurdles. First, existing evidence relies on retrospective cohorts with small sample sizes, carrying risks of confounding bias and statistical overfitting. Second, the human microbiome is highly susceptible to exogenous factors such as daily diet, geographic location, and antibiotic exposure; this baseline noise may obscure genuine signals. Technologically, globally standardized multi-omics analytical pipelines are still lacking; particularly for low-biomass samples like the skin or intratumoral microbiome, the exceedingly high host DNA background and risk of exogenous DNA contamination undermine analytical reproducibility (Bullman, 2023). To bridge this gap, future validation studies should strive to avoid purely descriptive associations and adopt rigorous experimental designs. To translate from candidate signals into actionable clinical tools, MHIBs must undergo rigorous validation conforming to regulatory frameworks, such as the FDA’s BEST (Biomarkers, EndpointS, and other Tools) guidelines. This requires analytical validation of multi-omics assays, followed by clinical validation and demonstration of clinical utility in large, independent prospective cohorts. A seminal example is the 2026 analysis of the global CheckMate 915 clinical trial, which demonstrated that when geographic and compositional variability were rigorously controlled, baseline gut microbial “fingerprints”(characterized by specific abundances of *Eubacterium*, *Ruminococcus*, and *Firmicutes*) could predict melanoma recurrence following adjuvant ICIs therapy with up to 94% accuracy (Usyk et al., 2026).

***Key Takeaways for Section 2:** Current evidence suggests that the gut and skin microbiota may bidirectionally influence melanoma initiation, progression, and immunity. These interactions involve immune cell education, metabolic signaling (e.g., SCFAs, formate, and secondary bile acids), and host physiological axes such as aging, endocrine signaling, and the UV resistome. Additionally, mechanism-driven MHIBs offer a proposed functional framework for patient stratification, which requires stringent clinical validation.*

## 3 Gut microbiota-mediated regulation of melanoma therapy

**Figure 2:**
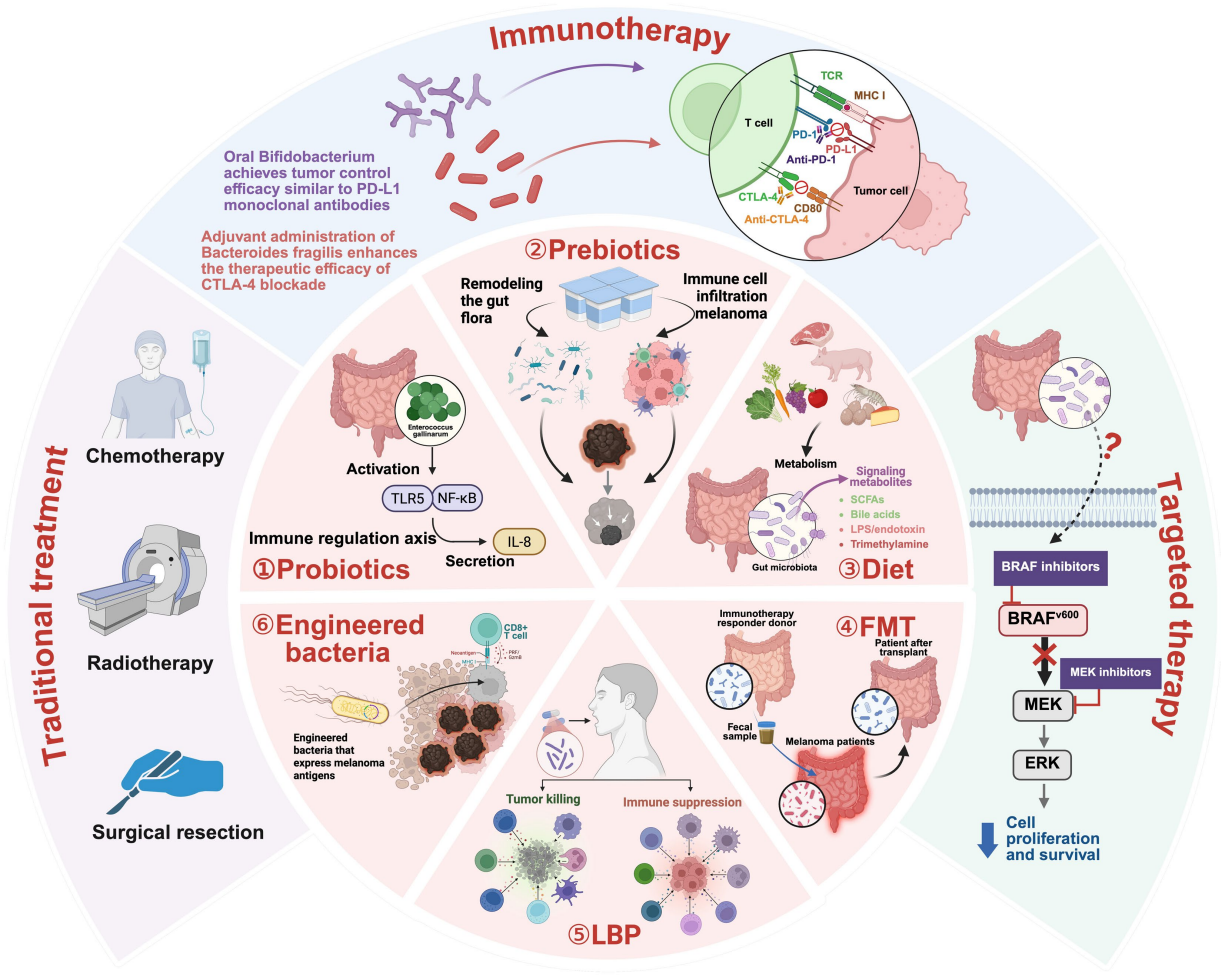
Melanoma therapy and microbiota-mediated modulation. This schema illustrates the regulatory effects of the gut and skin microbiota on the efficacy, therapeutic resistance and immune-related adverse events of mainstream melanoma treatments including immunotherapy, targeted therapy, chemotherapy and radiotherapy, and summarizes six core microbiota-targeted interventional strategies (probiotics, prebiotics, dietary interventions, fecal microbiota transplantation, live biotherapeutic products and engineered bacteria) for optimizing melanoma clinical treatment outcomes. Created with BioRender.com.

Accumulating evidence has demonstrated that the gut and skin microbiota are critical modulators of the therapeutic efficacy, treatment resistance and toxic reactions of nearly all mainstream therapeutic regimens for melanoma, from ICIs to conventional treatments. Meanwhile, microbiota-targeted interventions have emerged as promising adjunctive strategies to optimize therapeutic outcomes for melanoma patients. Figure 2. systematically summarizes the modulatory effects of the microbiota on different therapeutic modalities for melanoma, including immunotherapy, targeted therapy, chemotherapy, radiotherapy and surgical resection, and provides a structured overview of the six core microbiota-centered interventional strategies, namely probiotics, prebiotics, dietary interventions, FMT, live biotherapeutic products (LBPs) and engineered bacteria.

### 3.1 ICIs

ICIs are a class of monoclonal antibodies targeting immune checkpoint proteins, designed to restore the capacity of T cells to recognize and eliminate tumor cells (Bagchi et al., 2021). Anti-PD-1 and anti-CTLA-4 antibodies have become the first-line standard of care for advanced or metastatic melanoma (Klobuch et al., 2024), and significantly prolong progression-free survival (PFS) and overall survival (OS) (Robert et al., 2020). However, the clinical application of ICIs remains constrained by several key limitations. First, a small subset of melanoma patients exhibit primary non-response to the treatment; second, acquired resistance may emerge with long-term ICIs administration (Lim et al., 2023); third, treatment can induce irAEs (Reschke et al., 2025).

The gut microbiota modulates the response of melanoma to PD-1/PD-L1 therapy primarily through two distinct pathways. First, the antigen-specific pathway, in which shared epitopes between microbial and tumor antigens may trigger cross-reactive immune responses (Liu et al., 2025). Second, the antigen-independent pathway, whereby microbiota-derived metabolites shape the systemic and local tumor immune microenvironment via the systemic circulation (Perl et al., 2025). Inosine, in synergy with IFN-γ, drives Th1 cell differentiation and CD8^+^ T cell proliferation, thereby further augmenting the magnitude of anti-tumor immunity (Mager et al., 2020). The abundance of beneficial bacterial genera including *Akkermansia*, *Bifidobacterium* and *Faecalibacterium* is positively correlated with therapeutic response. These bacteria not only maintain the integrity of the intestinal mucosal barrier and alleviate systemic inflammation, but also modulate the balance of MDSCs, Tregs and M1/M2 macrophage polarization via their metabolites, thereby reversing the immunosuppressive TME (Blake et al., 2024). The anti-tumor activity of anti-CTLA-4 therapy has also been shown to depend on gut microbiota in preclinical models (Vétizou et al., 2015). Studies have revealed that CTLA-4 antibody-mediated immunotherapy may also induce colitis. This pathological response involves the unrestrained activation of CD4^+^ T cells and the depletion of intestinal Tregs. Removal of the Fc domain of the CTLA-4 antibody can attenuate the development of colitis while preserving its anti-tumor efficacy (Lo et al., 2024).

Accumulating evidence indicates that the gut microbiota is associated with ICIs efficacy and irAE pathogenesis. In murine melanoma models, oral Bifidobacterium improved tumor control to a degree comparable with PD-L1 blockade, and the combination produced stronger tumor inhibition (Sivan et al., 2015). *Enterococcus faecalis* enhances its immunogenicity through peptidoglycan remodeling, thereby promoting CD8^+^ T cell activation and boosting the therapeutic efficacy of ICIs (Griffin et al., 2021). Melanoma patients who respond to PD-1 blockade therapy harbor a significantly higher abundance of *Ruminococcus* species in the gut (Gopalakrishnan et al., 2018). Intratumoral bacteria in melanoma, such as *Burkholderia cepacia*, *Bacillus megaterium* and *Corynebacterium kroppenstedtii*, act synergistically with anti-PD-1 therapy to inhibit tumor growth (Chen J. et al., 2025). *Bacteroides* species can also downregulate the expression of PD-L2 and its ligand RGMb in the intestinal tract and tumor tissues, and enhance the efficacy of ICIs by modulating non-PD-1/PD-L1 signaling pathways (Park et al., 2023). Distinct microbial signatures are associated with the clinical efficacy of ICIs therapy and the development of specific adverse events in melanoma patients. Compared with patients without adverse events, those with irAEs exhibit microbial dysbiosis (Gao et al., 2025). In murine models, intestinal *Bifidobacterium* increases Treg abundance, and *Lactobacillus reuteri* reduces group 3 innate lymphoid cells (ILC3s), thereby alleviating ICI-induced colitis (Wang et al., 2019).

Extratumoral immune activation is critical for the intratumoral immune response (Franken et al., 2024; Wang K. et al., 2024). In the setting of combined immune checkpoint blockade (CICB) therapy, intestinal Bacteroides and IL-1β can serve as biomarkers for predicting adverse events. This study provides a fundamental basis for guiding CICB therapy and reducing the incidence of adverse events in patients with cancer (Andrews et al., 2021).

Antibiotics impair the response rate to immunotherapy and patient survival outcomes by disrupting gut microbiota diversity and homeostasis. Antibiotic administration prior to immunotherapy perturbs the homeostasis of the commensal gut microbiota, leading to primary resistance to immunotherapy and thereby compromising the anti-tumor immune response (Sivan et al., 2015; Gopalakrishnan et al., 2018; Routy et al., 2018; Pinato et al., 2019). Antibiotic exposure within 3 months before treatment initiation significantly reduces the 2-year OS rate in patients with stage III/IV melanoma, with penicillins, cephalosporins and fluoroquinolones conferring the highest risk. In contrast, antibiotic administration after the initiation of immunotherapy has no statistically significant impact on treatment outcomes (Pinato et al., 2019). Because antibiotics are often prescribed for infections or complications, indication bias and timing must be considered when interpreting these associations.

To date, most studies investigating the association between the gut microbiome and immunotherapy response are based on pre-treatment baseline data, while longitudinal studies conducted over the treatment course remain scarce. The response to subsequent anti-PD-1 therapy in melanoma patients with prior anti-CTLA-4 exposure is significantly associated with higher tumor mutational burden (TMB), activation of inflammatory signaling pathways, and cell cycle alterations. Furthermore, this prior treatment history is also linked to molecular features including enhanced intratumoral immune infiltration and gene mutations, and can be leveraged to improve the accuracy of predictive models for anti-PD-1 treatment response (Campbell et al., 2023). In addition to intestinal bacteria, fungi also harbor independent prognostic value for immunotherapy in melanoma patients. Patients with melanoma present with higher fecal abundance of fungal species including *Candida albicans*, *Candida dubliniensis* and *Neurospora crassa*, alongside lower abundance of beneficial fungi such as *Saccharomyces cerevisiae* and *Sporopachydermia hansenii*.

For ICIs, the evidence base includes melanoma-specific human cohorts and early interventional trials, but most taxa-response associations remain vulnerable to confounding by diet, antibiotics, PPIs, geography, treatment line, and sampling methods. Longitudinal sampling and prospective validation are needed before clinical deployment.

### 3.2 Crosstalk between the gut microbiota and targeted therapy

The molecular driving mechanisms of melanoma have been progressively elucidated, with the most prevalent driver mutations including BRAF, NRAS, and KIT (Zhang et al., 2023). These mutations drive uncontrolled cell growth, and promote the proliferation and survival of tumor cells via the aberrant activation of MAPK and other signaling pathways (Guo et al., 2020). BRAFV600 mutations account for approximately 50% of all melanoma cases, making it the most pivotal therapeutic target (Richtig et al., 2017). Combination therapy with BRAF/MEK inhibitors is the first-line standard of care for patients with BRAF-mutated advanced melanoma (Tian et al., 2023; Shi et al., 2024). In addition, KIT and NRAS mutations, as well as NTRK fusion alterations, are detected in a small subset of patients (Curti and Faries, 2021; Zhang et al., 2023; Theik et al., 2024; Lu et al., 2025). Overall, targeted therapy has dramatically improved the clinical outcomes of patients with melanoma, yet drug resistance and interindividual variability in treatment response remain the major challenges in current clinical practice. Beyond the intrinsic genomic features of tumor cells, the gut microbiota has emerged as a critical modulator of therapeutic response and treatment-related toxicity in recent years.

The composition of the gut microbiota is closely correlated with patient response to BRAF/MEK inhibitors, and specific microbial communities may modulate the depth and tolerability of targeted therapy response through immunomodulatory mechanisms. Patients with partial response (PR) have a gut microbiota enriched for *Lachnospiraceae*, *Coriobacteriaceae* and *Adlercreutzia* at baseline, while those with complete response (CR) exhibit enrichment of *Prevotellaceae*, *Cerasicoccaceae* and *Lawsonia*. *Oscillospira* is enriched in melanoma patients with moderate to severe treatment-related adverse events (TRAE), suggesting a potential association between this genus and inflammatory toxic reactions during targeted therapy (Guardamagna et al., 2025). Transcriptomic analyses reveal that patients harboring these favorable microbial profiles concurrently exhibit upregulated antigen-presentation genes (e.g., TAP1, PSMB8) and downregulated immunosuppressive markers (e.g., LAG3, CD36) in the tumor microenvironment or peripheral blood (Guardamagna et al., 2025). The reported temporal stability of the gut microbiota suggests that baseline microbial signatures may have predictive value, but the small sample sizes and observational design mean that probiotics, prebiotics, or FMT should not yet be considered validated strategies for optimizing targeted therapy. Current findings are best interpreted as preliminary evidence for a microbiota-immunity-targeted therapy network (Guardamagna et al., 2025).

Evidence for targeted therapy is weaker than for ICIs and currently rests mainly on small observational cohorts. Baseline microbial signatures should be considered candidate predictors, not validated tools for choosing BRAF/MEK inhibitor regimens.

### 3.3 Microbiota-mediated modulation of conventional therapies

Surgical resection is the primary curative treatment for early-stage melanoma (Schadendorf et al., 2015). For patients with locally advanced or metastatic disease, combined radiotherapy and/or chemotherapy is frequently required as an adjuvant therapeutic strategy (Testori et al., 2019). The adverse effects of radiotherapy can be categorized into acute toxicities and late toxicities (Verginadis et al., 2025). Chemotherapy was once widely used for the treatment of advanced melanoma, with commonly used agents including albumin-bound paclitaxel (nab-paclitaxel) (Hersh et al., 2015) and carboplatin (Hauschild et al., 2009), yet it is associated with a high incidence of adverse reactions (Lustberg et al., 2023). In addition, local treatment strategies such as intratumoral injection of the oncolytic virus T-Vec have also demonstrated modest anti-tumor activity in selected cases (Pavlick et al., 2023).

The gut microbiota affect the therapeutic efficacy of chemotherapy or radiotherapy in patients with melanoma, but such evidence remains limited. Therefore, most of the evidence discussed should be regarded as indirect, although it may still provide useful reference value. Mechanisms revealed by cross-cancer studies may have broader relevance, but they do not constitute melanoma-specific evidence. These mechanisms include microbiota-mediated drug metabolism, microbiota-derived metabolites that alter treatment sensitivity, and microbiota-associated mucosal or cutaneous toxicities. In other malignancies, specific microbiota-derived metabolites and intratumoral bacteria have been shown to modulate the efficacy of chemotherapy and radiotherapy—for instance, by altering DNA repair mechanisms or inducing chemoresistant phenotypes (Colbert et al., 2023; Teng et al., 2023; Tintelnot et al., 2023). However, given the unique immune contexture of melanoma, these diverse pan-cancer mechanisms cannot be directly extrapolated and require disease-specific validation.

Melanoma differs substantially from the above cancer types in its tissue of origin, immune contexture, clinical treatment regimens, and the roles of skin and intratumoral microbial communities. Melanoma-specific data indicate that the intratumour microbiome is associated with cytotoxic CD8^+^ T cell infiltration and patient survival in cutaneous melanoma (Zhu et al., 2021). In parallel, preclinical studies have shown that engineered skin commensals, particularly antigen-expressing *Staphylococcus epidermidis*, can elicit tumor-specific T cell responses and suppress melanoma growth in murine models (Chen et al., 2023). Nevertheless, these findings do not prove that microbiota-directed interventions can improve the efficacy of conventional chemotherapy or radiotherapy in patients with melanoma. Future studies should use prospective sampling to evaluate dynamic changes in the microbiota before and after surgery, radiotherapy, chemotherapy, and intralesional therapy, while comprehensively accounting for diet, antibiotics, corticosteroids, tumor burden, and prior immunotherapy.

The evidence level for conventional therapies is low to moderate: mechanistic support is strongest in non-melanoma preclinical or translational studies, while melanoma-specific clinical evidence remains limited and largely associative. Consequently, microbiome-based intervention during conventional melanoma treatment should be presented as a research direction, not a validated clinical strategy.

***Key Takeaways for Section 3:** Gut microbial profiles are closely associated with the efficacy and toxicity of ICIs and targeted therapies in melanoma. Specific commensal taxa and their metabolites may augment anti-tumor immunity and improve treatment responses. Conversely, antibiotic-induced dysbiosis impairs treatment outcomes, highlighting the need to preserve microbial homeostasis during systemic therapy. Evidence regarding conventional therapies remains primarily indirect and requires melanoma-specific validation.*

## 4 Clinical advances in microbiota-based interventions

### 4.1 Prebiotics and dietary interventions

Prebiotics are defined as compounds or ingredients that are utilized by the host microbiota to confer health or performance benefits (Deehan et al., 2024). Prebiotics are generally recognized as safe at conventional intake doses. Not all dietary fibers qualify as prebiotics, but prebiotics typically fall under the category of dietary fibers. In murine melanoma models, mucin and inulin altered gut microbial composition and enhanced anti-tumor immune infiltration; inulin also delayed acquired resistance to MEK inhibition in mice (Li Y. et al., 2020). These findings support prebiotics as candidate adjuvant interventions but do not establish clinical efficacy in patients with melanoma.

Other diet-derived or microbiota-modulated metabolites may also influence anti-tumor immunity, although the evidence is heterogeneous. For instance, dietary galactose has been shown to reprogram hepatocyte metabolism, which in turn upregulates insulin-like growth factor-binding protein 1 (IGFBP-1) and enhances CD8+ T cell function in pan-cancer models. However, its specific therapeutic efficacy in melanoma remains to be established (Du et al., 2025). Due to their low absorption efficiency in the small intestine, most dietary polyphenols reach the colon intact, where they are metabolized by the microbiota into bioactive compounds. For example, chestnutin, a polyphenol derivative, has been shown to alter gut microbial composition and elevate taurine-conjugated bile acids. In preclinical models, this metabolic shift optimizes immune cell ratios within the tumor microenvironment, thereby augmenting anti-tumor immunity and mitigating resistance to anti-PD-1 therapy (Cho et al., 2024).

Among human data, the clearest melanoma-relevant signal comes from dietary fiber and probiotic supplement use in patients receiving immune checkpoint blockade. Higher dietary fiber intake was associated with longer progression-free survival in 128 patients treated with ICIs, whereas commercially available probiotic use was associated with lower alpha diversity in patients; in parallel preclinical melanoma models, probiotic administration actively impaired anti-PD-L1 efficacy and reduced tumor-infiltrating T cells (Spencer et al., 2021). Fermented-food diets can increase gut microbial diversity and reduce inflammatory markers in healthy adults, but this evidence is not melanoma-specific (Wastyk et al., 2021). Therefore, fiber-rich and plant-rich dietary patterns are reasonable candidates for prospective testing, whereas high-salt dietary intervention should not be translated into a general recommendation for melanoma patients because of limited oncology evidence (Lisowski et al., 2025).

Additional nutritional strategies have been explored in preclinical or early clinical settings. Diet-gut microbiota-liver crosstalk may influence drug efficacy; for example, in murine cancer models, purified low-phytochemical diets, including ketogenic or high-carbohydrate formulations, enhanced PI3K inhibitor activity through phytochemical–microbiome–liver drug-metabolism interactions (Roichman et al., 2025). A serine/glycine-free diet reduced circulating serine and glycine, inhibited tumor growth, and promoted CD8^+^ T cell infiltration and activation in the TME; a phase I trial supported short-term safety and systemic immunomodulatory capacity (Tong et al., 2024). In melanoma, SCD-targeted dietary intervention should also be framed cautiously. Although SCD-deficient melanoma may be refractory to SCD inhibition, PTEN-wild-type/SCD-retained melanoma showed sensitivity to SCD inhibition. In preclinical melanoma, combining an SCD inhibitor with an isocaloric low-oleic-acid diet reduced intratumoral monounsaturated fatty acids and suppressed melanoma growth, with additional anti-tumor effects when combined with anti-PD-1 therapy (Oatman et al., 2024). Overall, calorie restriction, fasting-mimicking diets, ketogenic diets, protein restriction, and high-fiber diets affect cancer biology through metabolic reprogramming, TME remodeling, and microbiota changes, but most have not been validated in melanoma-specific randomized trials (Xiao Y. L. et al., 2024).

Nutritional and prebiotic interventions should currently be framed as candidate supportive strategies rather than approved melanoma therapies. Their clinical evaluation is limited by interindividual microbiome variability, baseline diet, supplement use. Methodological factors, including dietary questionnaires, sample collection, storage, DNA extraction, sequencing platform, can also generate non-comparable microbial signatures. Future studies should use standardized dietary assessment, longitudinal microbiome sampling, STORMS-compliant reporting, pre-specified endpoints, external validation cohorts, and long-term safety monitoring before microbiome-guided dietary interventions are promoted for routine melanoma care (Mirzayi et al., 2021; Lee et al., 2022).

### 4.2 Cancer microbiota-centered interventions

FMT is a whole-community microbiome intervention with established value in recurrent *Clostridioides difficile* infection, but its role in oncology remains investigational. Its rationale is to introduce donor-derived microbial communities that may reshape a dysregulated intestinal ecosystem. In melanoma, early trials have tested whether FMT can improve response to immune checkpoint blockade in patients with anti-PD-1-refractory disease (Baruch et al., 2021; Davar et al., 2021). In one single-arm study, FMT combined with pembrolizumab produced an objective response rate of approximately 20%, with durable stable disease in an additional subset of patients (Davar et al., 2021). In another phase I study of 10 patients with refractory metastatic melanoma, FMT from complete-response donors followed by anti-PD-1 reinduction produced clinical responses in three patients (Baruch et al., 2021). Recent long-term data further support this potential; the final results of the MIMic phase 1 trial demonstrated that FMT in combination with anti-PD-1 therapy safely improved clinical outcomes and overall survival in patients with advanced melanoma at a follow-up of more than 3 years (Hadi et al., 2025). These findings provide proof-of-concept evidence, but they do not show that FMT reliably reverses acquired resistance in all patients.

The variability of FMT outcomes is a major translational barrier. Donor selection, recipient baseline microbiota, host immune status, antibiotic exposure, stool preparation, route of administration, dosing schedule, and engraftment efficiency can all influence outcomes (Porcari et al., 2023). Donor screening must exclude pathogens, including multidrug-resistant organisms, and the eligibility rate of candidate donors can be low (Ng et al., 2024). The risk of microbial mismatch should also be considered: transfer of large-intestinal fecal microbiota into the small intestine can lead to aberrant colonization, bile-acid imbalance, abnormal lipid profiles, and systemic immune activation in translational contexts (DeLeon et al., 2025). Strain-level studies further suggest that engraftment is shaped by both donor-recipient complementarity and recipient ecological niches rather than by donor quality alone (Schmidt et al., 2022).

Safety and regulation are equally important. Regulatory approval of fecal microbiota products for recurrent *C. difficile* infection, such as oral SER-109/Vowst, demonstrates that microbiome-based products can be developed under defined indications, but this should not be equated with approval for melanoma or other cancers (Baruch et al., 2021; Carvalho, 2023). FDA safety communications have also emphasized the risk of transmitting pathogenic bacteria, including multidrug-resistant organisms, through FMT products. Therefore, oncology FMT studies require rigorous donor screening, adverse-event surveillance, product traceability, standardized manufacturing or processing, and clear regulatory pathways (Cammarota et al., 2019).

LBPs represent a more defined approach than whole-community FMT. LBPs are biological products containing live organisms that are intended to prevent, treat, or cure disease; they can include single-strain, multi-strain, or engineered microbial preparations (Frutos-Grilo et al., 2024). The LBP MRx0518, developed from *Enterococcus gallinarum*, has been investigated in combination with pembrolizumab in solid tumors. Its proposed mechanism involves flagellin-mediated activation of TLR5 and NF-κB signaling, with IL-8 induction in HT29-MTX cells (Lauté-Caly et al., 2019). VE800, an 11-strain consortium isolated from healthy human donors, enhanced anti-PD-1 efficacy in preclinical models (Tanoue et al., 2019). These examples support biological plausibility, but melanoma-specific efficacy and optimal dosing require prospective clinical validation. In addition, LBP development must address strain identity, genetic stability, purity, potency, manufacturing consistency, containment, and investigational new drug requirements (Cordaillat-Simmons et al., 2020).

Probiotics should not be treated as uniformly beneficial microbiota therapies. Their effects depend on strain identity, dose, the host’s baseline microbiota, immune status (Hill et al., 2014). Reuterin secreted by *Lactobacillus reuteri*, when encapsulated in covalent organic frameworks, showed anti-tumor activity in experimental models (Zhang et al., 2025). In contrast, certain probiotics, including *Bifidobacterium longum* or *Lactobacillus rhamnosus*, impaired anti-PD-L1 response in melanoma models by reducing tumor-infiltrating IFN-γ^+^ CD8^+^ T cells and accelerating tumor growth (Spencer et al., 2021). In patients with advanced melanoma receiving ICIs, over-the-counter probiotic use was associated with less favorable outcomes in some analyses, whereas higher dietary fiber intake was associated with improved ICIs response (Zitvogel et al., 2022). Thus, probiotic use during melanoma immunotherapy should be considered context-dependent and should not be recommended as a generic supportive intervention without strain-specific evidence.

Engineered bacteria provide a more programmable but less clinically mature strategy. Skin commensal *Staphylococcus epidermidis* engineered to express melanoma antigens induced antigen-specific CD8^+^ T cell responses and suppressed melanoma growth in murine models, with stronger activity when combined with ICIs (Chen et al., 2023). Engineered *Escherichia coli* Nissle 1917 enhanced antigen expression and cytosolic delivery, promoted antigen presentation and CD8^+^ T cell activation, inhibited local tumor growth, and induced regression of distant tumors in advanced metastatic models (Redenti et al., 2024). Designer Bacteria 1 (DB1), which exploits IL-10 receptor hysteresis within the TME, represents another experimental approach to sustain anti-tumor activity (Chang et al., 2025). However, these strategies remain primarily preclinical. Translation will require evidence on biosafety, horizontal gene transfer, persistence, clearance, immune toxicity, manufacturing reproducibility, and regulatory oversight.

Microbiota-centered interventions in melanoma currently range from early human proof-of-concept studies to preclinical engineering platforms. The evidence is strongest for associations between the gut microbiome and ICIs response, moderate for early FMT plus anti-PD-1 rechallenge, and still preliminary for probiotics, defined LBPs, and engineered bacteria. Accordingly, FMT, LBPs, probiotics, and engineered bacteria should be described as investigational strategies rather than routine components of melanoma care (Gopalakrishnan et al., 2018; Spencer et al., 2021).

Microbiota-centered interventions, including dietary modulation, prebiotics, FMT, and engineered bacteria, represent investigational strategies to overcome treatment resistance. Early clinical data for FMT combined with anti-PD-1 therapy show promise in a subset of patients. However, substantial translational barriers remain. These include unpredictable donor strain engraftment, microbial mismatch, regulatory hurdles, and potential safety risks regarding pathogen transmission.

## 5 Evaluation of current evidence

Although the gut-skin axis shows potential in regulating melanoma development and reshaping ICIs responses, the field is currently transitioning from phenotypic observation to mechanistic validation. Most human cohort studies are cross-sectional or retrospective. They infer statistical correlations by comparing baseline microbial abundance between responders and non-responders (Gopalakrishnan et al., 2018). However, a significant gap remains between these correlations and true biological causality. A fundamental challenge in the field is distinguishing causality from correlation. It remains actively debated whether microbial dysbiosis mechanistically drives immunotherapy resistance or primarily represents a “passenger” phenomenon—an epiphenomenon reflecting advancing tumor burden or systemic inflammation (Sepich-Poore et al., 2021). Consequently, cross-sectional statistical associations should be interpreted cautiously and not definitively equated with causal determinants.

For mechanistic exploration, current research relies heavily on specific pathogen-free (SPF) mice, germ-free mice, and murine FMT (Sivan et al., 2015). This dependence on preclinical models faces skepticism due to translational barriers (Hugenholtz and de Vos, 2018). Fundamental differences exist between murine and human immune systems and gut anatomy. Standardized laboratory mice lack complex antigen exposure. They remain in a “naive” immune state and often exhibit more acute immune responses than humans (Pitt et al., 2016). Furthermore, human obligate anaerobes face extreme colonization resistance in mice, leading to distorted reconstructed microbiomes (Chen-Liaw et al., 2025). Additionally, mechanistic validation frequently utilizes high-dose single-strain oral gavage, which is difficult to replicate in the complex human environment. Consequently, extrapolating mechanistic data from these murine models to human clinical practice carries a high risk of false positives (Walter et al., 2020).

In human cohort studies, small sample sizes and methodological variability in multi-omics analyses undermine the generalizability of existing conclusions. Many pioneering studies involve only dozens to over a hundred patients (Frankel et al., 2017). When analyzing compositional microbiome data, these small sample sizes easily trigger false positives from multiple hypothesis testing and model overfitting (Wirbel et al., 2021). Consequently, the predictive accuracy of models trained on a single cohort drops sharply during cross-cohort validation, yielding extremely low biomarker overlap (Lee et al., 2022). This raises concerns that current findings may merely reflect localized effects driven by specific genetic backgrounds and medical practices (Pietrzak et al., 2022). Furthermore, sample collection, storage, and the use of different DNA extraction kits introduce “amplification bias”(Vandeputte et al., 2017). Early studies also heavily relied on 16S rRNA sequencing, which provides limited resolution (Jovel et al., 2016). Notably, when analyzing low-biomass samples like skin or intratumoral tissues, environmental contamination and host DNA backgrounds pose severe technical challenges to extracting genuine microbial signals (Gihawi et al., 2023).

Clinical confounding factors implicitly interfere with studies and can easily lead to attribution fallacies. The human microbiome is a highly open system. Inter-patient differences in macronutrient intake, such as dietary fiber, can profoundly alter the intestinal metabolite pool (Rothschild et al., 2018). Regarding dietary data collection, existing studies often rely on subjective food frequency questionnaires (FFQs). These are difficult to quantify precisely and cannot exclude recall bias (Spencer et al., 2021). Meanwhile, although the destructive impact of broad-spectrum antibiotics on the microbiota is widely documented (Derosa et al., 2018), proton pump inhibitors (PPIs) and other common medications can also independently alter microbial composition (Cortellini et al., 2020). Without rigorous adjustment, attributing microbial dysbiosis simply to melanoma progression or using it as an independent efficacy predictor lacks scientific stringency. More alarmingly, negative-result studies indicate that the blind supplementation of unmatched over-the-counter (OTC) probiotics provides no benefit. Instead, it may reduce intestinal diversity and interfere with ICI efficacy (Zitvogel et al., 2022).

Similar bottlenecks exist in clinical interventions and cross-cancer evidence extrapolation. Early clinical trials of FMT combined with ICIs have demonstrated preliminary potential to reverse drug resistance (Davar et al., 2021). However, these are mostly single-arm, small-sample Phase I/II exploratory studies that lack large-scale randomized controlled trials (RCTs). Furthermore, donor strain engraftment efficiency in recipients fluctuates significantly (Baruch et al., 2021). The potential risks of such broad ecological replacement concerning long-term safety and the induction of systemic irAEs remain incompletely resolved (Routy et al., 2023). Due to the scarcity of melanoma-specific microbiome clinical data, some mechanistic discussions in this review borrow extrapolated evidence from NSCLC and gastrointestinal tumors (Elkrief et al., 2019). The uniqueness of melanoma includes an exceptionally high TMB, a distant immune network dependent on skin-homing receptors, and a distinct skin microbiome background (L'Orphelin et al., 2025). Currently, candidate biomarkers extrapolated from other solid tumors (e.g., specific secondary bile acids or MAdCAM-1) hold only hypothesis-generating value for mechanistic exploration (Jin et al., 2025).

To objectively reflect current research progress and clarify the differences in evidence weight among “clinical observation,” “animal mechanisms,” and “cross-cancer extrapolation,” we strictly categorized the core research evidence covered in this review. The evidence hierarchy, core findings, and translational limitations are summarized in Table 2.

**Table 2:** Summary of evidence categories for the gut-skin microbiome in melanoma research.

Evidence category	Core research and intervention	Key findings	Limitations and translational barriers	References
Level I evidence (human intervention: FMT)	Early-phase exploratory trials of FMT combined with ICIs	Reverses acquired anti-PD-1 resistance Promotes CD8+ T cell tumor infiltration Reshapes systemic inflammation	Lacks large-scale RCTs (small sample sizes) Lacks standardized donor and preparation criteria Highly variable engraftment rates; potential systemic irAE risks	Baruch et al. (2021), Davar et al. (2021)
Level II evidence (human observational cohorts)	Association between baseline gut microbiome and ICI efficacy	Identifies favorable taxa (e.g., Faecalibacterium) High abundance correlates with high ORR and prolonged PFS	Small sample sizes risk statistical overfitting 16S sequencing lacks strain-level resolution; host DNA limits low-biomass analysis High confounding (genetics, medical background) leads to poor cross-cohort reproducibility	Gopalakrishnan et al. (2018), Lee et al. (2022)
Level III evidence (epidemiology and confounders)	Impact of dietary fiber, probiotics, and medications on ICI efficacy	High dietary fiber extends PFS Broad-spectrum antibiotics impair antitumor immunity OTC probiotics can reduce diversity and hinder ICI efficacy	Food frequency questionnaires (FFQs) carry recall bias Difficult to fully adjust for concurrent medications (e.g., PPIs) High heterogeneity in commercial probiotic formulations	Spencer et al. (2021), Zitvogel et al. (2022)
Level IV evidence (preclinical models)	Microbiome mechanism validation in SPF or germ-free mice	Activates immune pathways (e.g., TLRs) Enhances DC antigen presentation and T cell cytotoxicity Suppresses murine tumor growth	Murine immune systems lack natural antigen exposure Significant human-mouse differences in gut anatomy and colonization resistance Extreme high-dose gavage limits human translatability (false-positive risk)	Sivan et al. (2015), Walter et al. (2020)
Extrapolated evidence (non-melanoma solid tumors)	Biomarker and mechanism discovery in other solid tumors (e.g., NSCLC)	Secondary bile acids antagonize AR to enhance immunity Dysbiosis downregulates MAdCAM-1	Major differences in TMB, TME, and microbiomes across cancer types Only holds hypothesis-generating value; lacks melanoma-specific cohort validation	Routy et al. (2018), Fidelle et al. (2023), Jin et al. (2025)

## 6 Discussion

The bidirectional gut-skin axis, which mediates crosstalk between the gut and skin commensal microbiota, is a core pathway regulating melanoma tumorigenesis, progression, immunotherapy response, and prognosis. Most previous studies have focused on the unidirectional effects of gut microbiota on melanoma, while neglecting the synergistic regulatory effects of the gut-skin axis and skin microbiota, leaving a gap in the systematic understanding of microbiota-driven melanoma pathogenesis. We have mapped the specific signature profiles of gut and skin microbiota in melanoma patients across different lesion benign/malignant status, clinical stages, molecular subtypes, ICIs response, and irAEs conditions. We have also constructed a mechanistic network of melanoma regulation by the gut-skin axis via six core axes: immune regulation, aging, endocrine signaling, metabolism, circadian rhythm, and ultraviolet radiation, and identified bidirectional gut-skin crosstalk as the core mediator of melanoma progression. Furthermore, we propose MHIBs and a multi-omics-based functional microbial typing framework, and systematically summarize six microbiota-targeted intervention strategies and their synergistic anti-tumor potential with ICIs. Against this background, the primary contribution of this review is not to establish a definitive dogma, but to systematically integrate dispersed evidence across the cutaneous and intestinal compartments to conceptualize a bidirectional “microbiota-gut-skin axis” working model. This framework differentiates itself from prior unidirectional gut-tumor reviews by explicitly incorporating systemic physiological and environmental variables as critical modulators.

Despite these advances, critical unresolved challenges remain in the field, which define key priorities for future research. At the mechanistic level, the causal link between bidirectional gut-skin axis crosstalk and melanoma remains poorly defined. Existing evidence is mostly derived from mouse models and retrospective association studies, with a lack of causal validation in human physiological contexts. The synergistic or antagonistic cross-talk between the six core regulatory axes, as well as the key microbial metabolites mediating gut-skin communication, have also not been fully elucidated. Future studies should prioritize causal validation using germ-free mice, patient-derived xenograft (PDX) models, and humanized models, combined with spatial multi-omics techniques to dissect the cross-regulatory network of the six core axes, with a focus on identifying key microbial metabolites and their specific host receptors that drive melanoma progression.

In terms of technical standards and tool development, key bottlenecks persist in melanoma microbiota research and engineered bacteria design. Skin microbiota samples have extremely low biomass, high host DNA content, and uncontrollable exogenous contamination risks. Current detection methods have limited ability to distinguish live and dead bacteria, and inconsistent sampling, sequencing, and analysis pipelines across studies lead to poor data comparability. Meanwhile, the generalizability and stability of our proposed MHIBs and microbial typing framework have only been validated in small retrospective cohorts. For engineered bacteria, existing strains face technical hurdles including low targeted delivery efficiency to melanoma lesions, poor colonization stability in the skin or gut, insufficient controllability of anti-tumor functional element expression, and off-target immunogenicity risks. Furthermore, the transition of MHIBs from candidate signals to actionable clinical tools requires strict adherence to formal regulatory frameworks, such as the FDA’s BEST guidelines. Validating these biomarkers demands rigorous analytical validation to ensure assay reproducibility and mitigate the risk of false discoveries inherent in low-biomass tumor microbiome studies. This must be followed by clinical validation and utility demonstration in large, prospective, multi-center cohorts.

For clinical translation, microbiota-centered interventions must navigate evolving regulatory landscapes and substantial safety concerns, including potential pathogen transmission during FMT. Furthermore, translation is complicated by inconsistent clinical outcomes. Recent observations indicate that indiscriminate over-the-counter probiotic supplementation may reduce microbial diversity and yield detrimental effects on ICIs efficacy. These negative findings underscore the inherent dangers of empirical microbiome manipulation without precise, strain-level functional validation. Future clinical translation should focus on personalized intervention strategies based on MHIBs and microbial typing, conduct multi-center RCTs to verify the efficacy and safety of different regimens in specific melanoma populations, establish standardized clinical protocols for microbiota intervention combined with ICIs, and develop melanoma-specific next-generation live biotherapeutic products including intelligent probiotics and engineered bacteria.

Collectively, research on the gut-skin axis microbiota has opened a new avenue for the precise diagnosis and treatment of melanoma. With the elucidation of causal mechanisms, improvement of technical systems, and advancement of clinical translation, targeted modulation of the microbiota-gut-skin axis may evolve from an investigational concept into a synergistic adjunct for comprehensive melanoma management. Ultimately, this approach aims to optimize patient stratification, minimize toxicity, and improve survival outcomes.
